# Place and Temporal Cues in Cochlear Implant Pitch and Melody Perception

**DOI:** 10.3389/fnins.2019.01266

**Published:** 2019-11-26

**Authors:** Brett A. Swanson, Vijay M. R. Marimuthu, Robert H. Mannell

**Affiliations:** ^1^Cochlear Ltd., Sydney, NSW, Australia; ^2^Department of Linguistics, Faculty of Human Sciences, Macquarie University, Sydney, NSW, Australia

**Keywords:** cochlear implant, pitch, melody, discrimination, sound coding

## Abstract

The present study compared pitch and melody perception using cochlear place of excitation and temporal cues in six adult nucleus cochlear implant (CI) recipients. The stimuli were synthesized tones presented through a loudspeaker, and recipients used the Advanced Combinational Encoder (ACE) sound coding strategy on their own sound processors. Three types of tones were used, denoted H3, H4, and P5. H3 tones were harmonic tones with fundamental frequencies in the range C3–C4 (131–262 Hz), providing temporal pitch cues alone. H4 tones were harmonic tones with fundamental frequencies in the range C4–C5 (262–523 Hz), providing a mixture of temporal and place cues. P5 tones were pure tones with fundamental frequencies in the range C5–C6 (523–1046 Hz), providing place pitch cues alone. Four experimental procedures were used: pitch discrimination, pitch ranking, backward modified melodies, and warped modified melodies. In each trial of the modified melodies tests, subjects heard a familiar melody and a version with modified pitch (in randomized order), and had to select the unmodified melody. In all four procedures, many scores were much lower than would be expected for normal hearing listeners, implying that the strength of the perceived pitch was weak. Discrimination and ranking with H3 and P5 tones was poor for two-semitone intervals, but near perfect for intervals of five semitones and larger. H4 tones provided the lowest group mean scores in all four procedures, with some pitch reversals observed in pitch ranking. Group mean scores for P5 tones (place cues alone) were at least as high as those for H3 tones (temporal cues alone). The relatively good scores on the melody tasks with P5 tones were surprising, given the lack of temporal cues, raising the possibility of musical pitch using place cues alone. However, the alternative possibility that the CI recipients perceived the place cues as brightness, rather than musical pitch *per se*, cannot be excluded. These findings show that pitch perception models need to incorporate neural place representations alongside temporal cues if they are to predict pitch and melody perception in the absence of temporal cues.

## Introduction

### Normal Hearing

For a pure tone in normal hearing, a place cue to pitch is provided by the location of the peak response on the basilar membrane, and a temporal cue to pitch is provided by neural phase locking, i.e., the neurons tend to fire in phase with the basilar membrane vibration (Moore, 1997; Oxenham, 2013). For a harmonic tone, the perceived pitch is equal to the fundamental frequency (F0), regardless of the amplitudes of the harmonics, even if there is no energy at F0 (a “missing fundamental”). The resolved harmonics are those lower harmonics which produce distinct peaks in the basilar membrane response. Each resolved harmonic provides a distinct place and temporal cue. The remaining (i.e., unresolved) harmonics do not provide a clear place cue (because a broad region of the cochlea is excited), but do provide a temporal cue (because the basilar membrane response is amplitude modulated at F0) (Plack and Oxenham, 2005).

Place cues to pitch in normal hearing are not very reliable: as the amplitude of a pure tone increases, the peak of basilar membrane excitation shifts basally, and the neural firing rate saturates over a region near the peak, yet the perceived pitch is relatively stable (Moore, 1997). Further evidence of the importance of temporal cues is that tones containing only unresolved harmonics do provide pitch percepts, albeit not as strong as that produced by resolved harmonics (Moore and Rosen, 1979; Houtsma and Smurzynski, 1990).

Timbre is the perceptual quality that allows two tones that have the same pitch, loudness, and duration to be distinguished. One aspect of timbre is brightness. Increasing the amplitudes of the high harmonics relative to the low harmonics increases the perceived brightness of a tone. Brightness can be ordered on a low-to-high scale, and the results of brightness ranking experiments can be predicted by the spectral centroid (“center of gravity”) of the tones (Plomp, 1976; Schubert and Wolfe, 2006). Multi-dimensional scaling experiments suggest that pitch and brightness are independent perceptual dimensions (Plomp, 1976).

A melody can be defined as a sequence of notes, typically varying in pitch and duration. To transpose a melody means to shift the entire melody up or down in pitch, adding (or subtracting) a constant number of semitones to each note. Transposing a melody does not change its identity; only the intervals (the differences in pitch from one note to the next) are important (Attneave and Olson, 1971). The contour of a melody is defined as the sequence of up or down changes in pitch, i.e., the direction of the steps, ignoring their size. Dowling and Fujitani (1971) showed that listeners have a good memory for the precise interval sizes of familiar melodies. However, distorted versions of familiar melodies that preserved the contour but changed the interval sizes could still be recognized reasonably well.

### Cochlear Implants

Cochlear implant (CI) temporal pitch cues can be investigated by stimulating a single electrode with a varying pulse rate. For pulse rates in the range of about 50–300 pulses per second, CI recipients can recognize melodies (Eddington et al., 1978; Pijl and Schwarz, 1995b), judge the size of presented musical intervals (Pijl and Schwarz, 1995b; McDermott and McKay, 1997; Pijl, 1997), and adjust pulse rates to produce a specified interval from either a fixed or randomized reference pulse rate, demonstrating an ability to transpose intervals (Pijl and Schwarz, 1995a; McDermott and McKay, 1997; Pijl, 1997). At these pulse rates, the neural firing is entrained to the stimulation pulses, i.e., the timing of neural firing is the same as the pulse timing (McKay et al., 1994). Similar pitch percepts are produced by varying the modulation frequency of an amplitude-modulated high rate pulse train (McKay et al., 1994; McDermott and McKay, 1997; Kong et al., 2009). The pitch is stronger for deeper modulation depths, and for shallow depths, the pitch may be higher than the modulation frequency (Vandali et al., 2013).

Cochlear implant place pitch cues can be investigated by varying the electrodes that are stimulated (Nelson et al., 1995). Several studies suggest that CI place pitch and temporal pitch are independent perceptual dimensions (Tong et al., 1983; McKay et al., 2000). The single CI recipient in McDermott and McKay (1997) could estimate a musical interval when two electrodes were stimulated in succession, but with little knowledge of the electrode placement, there was no objective way of determining the “correct” interval, and his estimates were more variable than those created when varying pulse rate on one electrode. McDermott and McKay (1994) found that sequential stimulation on two nearby electrodes evoked a place pitch percept that was intermediate to that of either electrode when stimulated by itself, and suggested that the percept depended on the centroid of the neural excitation pattern. Laneau et al. (2004) conducted a study in which four CI recipients pitch-ranked harmonic tones processed by the ACE strategy, using standard and alternative filter banks. In one condition, the filter envelopes were low-pass filtered to remove the amplitude modulation (temporal cues), leaving only place cues, and the results were predicted well by the centroid model.

There is an intriguing resemblance between the spectral centroid model for brightness in normal hearing and for place pitch in CI. The perceptual independence of temporal and place percepts in CI is also reminiscent of the independence of pitch and brightness in normal hearing. If CI place percepts were more akin to brightness than to musical pitch, then it would be expected that good scores could be obtained using CI place cues alone on discrimination and ranking tasks, but not on tasks that involve melodies (Moore and Carlyon, 2005). The present study investigated this hypothesis by comparing CI recipient performance on discrimination, ranking, and melody tasks, with stimuli that provided place cues alone or temporal cues alone.

In CI experiments that aim to independently manipulate temporal and place pitch cues, stimulus pulses are customarily delivered to a CI recipient under the control of a research interface. The present study also aims to demonstrate that CI temporal and place pitch cues can be studied by applying judiciously chosen audio signals to a recipient’s own sound processor. To this end, the results will be compared to those of our previous study (Marimuthu et al., 2016) investigating pulse rate cues to pitch in the same set of subjects, with stimuli delivered via a research interface. The present study also builds upon an earlier study (Swanson et al., 2009) in which melodies were presented by playing pure tones to the recipients’ processors.

## Materials and Methods

### Subjects

Six post-lingually-deafened adult CI recipients, with at least 1 year of implant usage, participated in the study. These were the same subjects as in our previous study on rate-pitch perception (Marimuthu et al., 2016).

### Cochlear Implant Signal Processing

**FIGURE 1:**
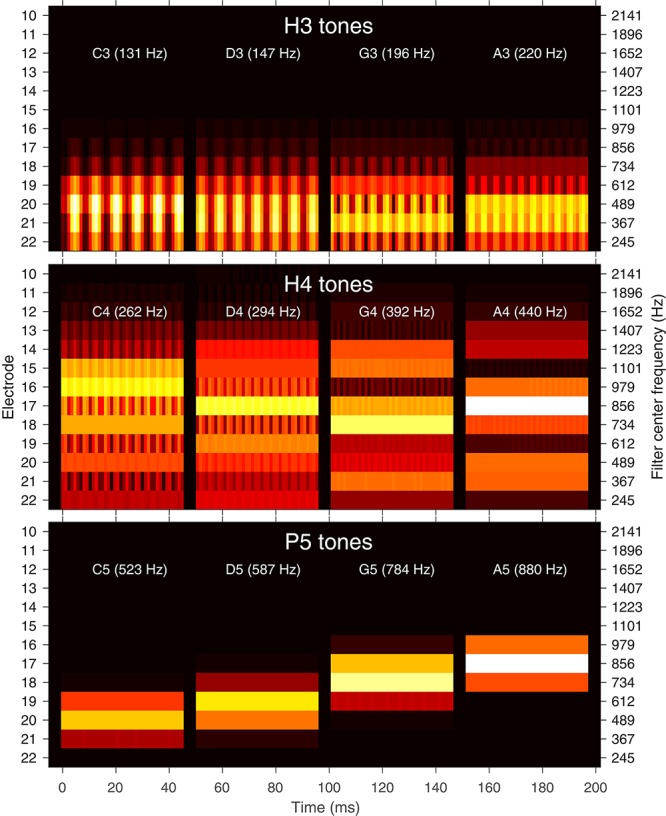
Filter bank envelopes (spectrograms) for each note. Amplitude is indicated by the color (black: zero, white: full scale). Electrodes in the Nucleus^®^ CI system are labeled from E22 (most apical) to E1 (most basal). E1–E9 had negligible energy for these tones and are not shown. The **center** frequencies of the corresponding filters are indicated on the **right** axis. A 45 ms excerpt of each of the four notes C, D, G, and A is shown (each note was 300 ms in duration with 50 ms rise and fall). Each note is also labeled with its fundamental frequency in hertz. **Top**: H3 tones (temporal cues only). **Middle**: H4 tones (temporal and place cues). **Bottom**: P5 tones (place cues only).

During the testing, all subjects used their own sound processor which implemented the Advanced Combinational Encoder (ACE) processing strategy (Vandali et al., 2000; Swanson et al., 2007). The ACE filter bank was based on a 128-point fast Fourier transform (FFT) with a Hann window. The audio sampling rate was 15,659 Hz, thus the FFT provided a bank of 64 filters with center frequencies spaced linearly at multiples of 122 Hz (Harris, 1982), and a 6 dB bandwidth of 245 Hz (two bins) (Harris, 1978). The FFT filters with center frequencies from 245 to 1101 Hz were allocated to the eight lowest frequency (most apical) electrodes (Figure 1). Wider filters for the more basal electrodes were formed by summing adjacent FFT bins. Each filter output sample was a complex number, and the envelope of each filter was calculated by taking the magnitude of these complex numbers; this is known as quadrature envelope detection (Swanson et al., 2007). All subjects used 22 electrodes, except for S4, who used 20 electrodes (E4 and E13 were deactivated). The eight lowest frequency filters were identical in all subjects.

After each FFT, a maxima-selection block examined each set of filter envelopes, and selected the eight channels with the largest amplitude for stimulation. Instantaneous non-linear compression was applied. Amplitudes corresponding to 65 dB SPL were mapped to the maximum comfortable current level (“C level”) of that channel. Amplitudes corresponding to 25 dB SPL were mapped to the threshold current level (“T level’), and lower amplitudes were discarded.

Subject S2 used a channel stimulation rate of 500 Hz, and the remaining subjects used 900 Hz.

### Stimulus Description

All the stimuli were synthesized at a sampling rate of 16 kHz, and presented via loudspeaker in a sound-treated room. Each note was 300 ms in duration with a smooth (sinusoidal-shaped) rise and fall time of 50 ms. The stimuli were presented at a comfortable loudness level. Three types of tones were used, here denoted H3, H4, and P5, as described in detail below.

**FIGURE 2:**
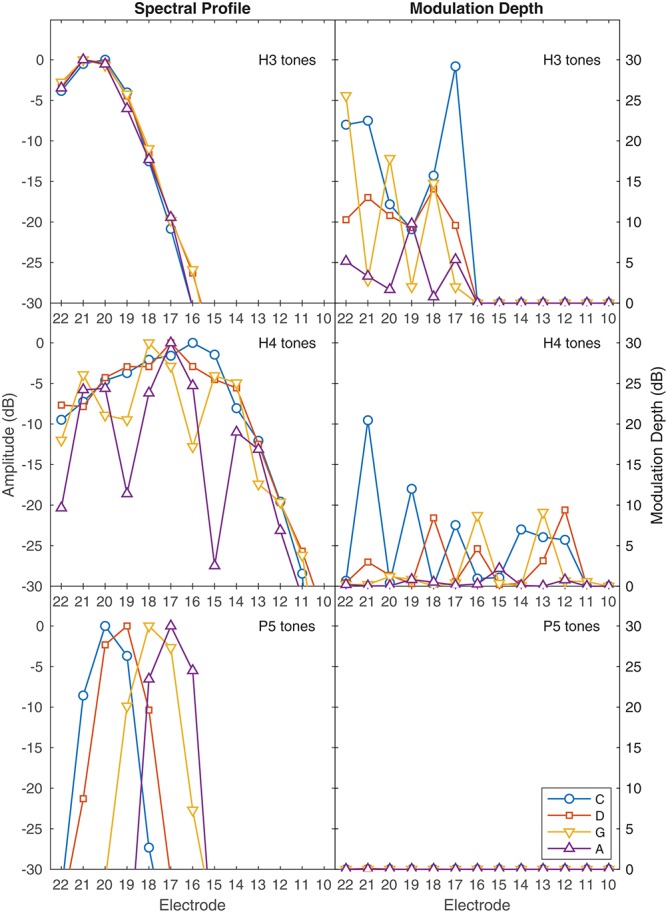
Representation of place and temporal cues in the ACE filter bank envelopes. The **left** set of panels shows the spectral profiles for each note, indicating the availability of place pitch cues. Each panel shows the peak amplitude in dB in each channel, corresponding to a vertical slice through the spectrograms in Figure 1. The **right** set of panels shows the amplitude modulation depth for each note, indicating the availability of temporal pitch cues. Each panel shows the modulation depth in dB in each channel. For those notes that had modulation, the modulation frequency was equal to the fundamental frequency of the note, as shown in the labels of Figure 1. The abscissa indicates the electrode number allocated to each filter; E1–E9 had negligible amplitude for these notes and are not shown. **Top panels**: H3 tones, where no harmonics were resolved, showing negligible differences in the spectral profile between notes, and deep modulation. **Middle panels**: H4 tones, spanning the range of fundamental frequencies from unresolved (C4) to resolved (A4) harmonics, with shallower modulation. **Bottom panels**: P5 tones, where each note produced a single peak in the spectral envelope, with no modulation.

**FIGURE 3:**
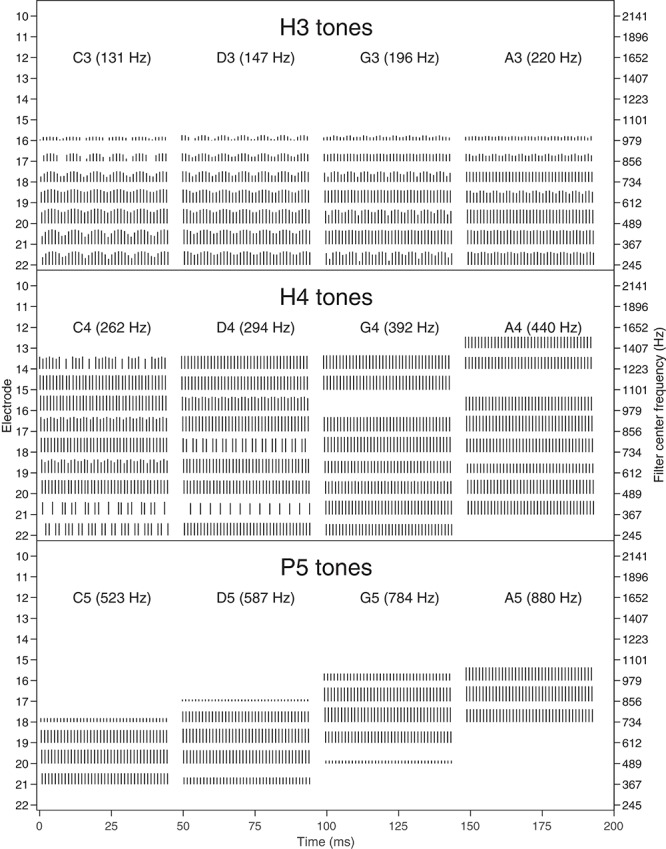
Pulse sequences (electrodograms) for each note. Each pulse is represented by a vertical line, with the horizontal position indicating the start time (onset) of the pulse, the vertical position indicating the electrode number (indicated on the **left** axis), and the height of the line representing the current level. E1–E9 had no pulses for these notes and are not shown. The **center** frequencies of the corresponding filters are indicated on the **right** axis. A 45 ms excerpt of each of the four notes is shown, as in Figure 1. **Top**: H3 tones (temporal cues only). **Middle**: H4 tones (temporal and place cues). **Bottom**: P5 tones (place cues only).

The stimuli are illustrated in several ways. Figure 1 shows the envelopes at the output of the ACE filter bank, plotted as 22-channel spectrograms. Figure 2 contains alternative representations of these envelopes to better visualize the place and temporal cues. The left set of panels in Figure 2 shows the spectral profiles (i.e., each corresponding to a vertical slice through the spectrograms of Figure 1), indicating the availability of place cues to pitch. The right set of panels in Figure 2 shows the modulation depth in each channel, indicating the availability of temporal cues to pitch. Lastly, Figure 3 shows the corresponding pulse sequences resulting from the ACE strategy with eight maxima and a channel stimulation rate of 900 Hz.

#### H3 Tones: Harmonic Tones in Octave 3

H3 tones were harmonic tones in octave 3, i.e., having F0s in the range from C3 to C4 (131–262 Hz). In synthesizing the H3 tones, harmonics were summed with zero phase, and with unity amplitude up to a corner frequency of C5 (523 Hz), and then with amplitude decreasing at -36 dB per octave up to an upper frequency of C6 (1046 Hz). This spectral shaping is visible in the spectrograms (Figure 1). The individual harmonics were not resolved by the ACE filter bank. As a result, the four notes (C3, D3, G3, A3) had the same spectral profile (Figure 2), and the resulting pulse sequences (Figure 3) activated the same set of electrodes (E14–E22), with the same peak amplitudes, and hence there were negligible place pitch cues.

Multiple harmonics fell into each ACE filter, resulting in envelopes with amplitude modulation at the fundamental frequency, clearly visible in the spectrograms (Figure 1). The corresponding pulse sequences (Figure 3) had current level modulation at F0, providing a temporal pitch cue. The salience of the temporal pitch cue is related to the modulation depth (Vandali et al., 2013). The modulation depth (Figure 2) differed across channels within one note, depending on the alignment of the harmonics to the filter center frequencies. The average modulation depth reduced as F0 increased, being deepest for C3 and shallowest for A3.

#### H4 Tones: Harmonic Tones in Octave 4

H4 tones were harmonic tones in octave 4, i.e., having F0s ranging from C4 to C5 (262–523 Hz). The H4 tones were synthesized in a similar manner to the H3 tones, except that the corner frequency was C6 (1046 Hz) and the upper frequency was C7 (2093 Hz). This spectral shaping is again visible in the spectrograms (Figure 1). As the fundamental frequency increased, the ACE filter bank progressively resolved the harmonics. For example, referring to Figure 2, the spectral profile of the note C4 reflected the overall spectral shaping; the harmonics were not resolved because their spacing (262 Hz) was comparable to the filter bandwidth (245 Hz). In contrast, the spectral profiles for notes G4 (392 Hz) and A4 (440 Hz) showed three distinct spectral peaks, corresponding to the first three harmonics being resolved.

The modulation depth (Figure 2) exhibited a complementary pattern: channels corresponding to peaks in the spectral profile had the least modulation. In the note C4, the first four harmonics had frequencies close to the center frequencies of the filters driving electrodes E22, E20, E18, and E16. Thus, those channels were dominated by a single harmonic and had negligible amplitude modulation. Conversely, electrodes E21, E19, and E17 responded to two harmonics, and were deeply modulated. As F0 increased, there were fewer channels that responded to two harmonics, and the modulation depth decreased. Note A4 had very little modulation. Figure 3 shows the corresponding pulse sequences. Thus, the H4 tones offered a mixture of temporal and place cues to pitch.

#### P5 Tones: Pure Tones in Octave 5

P5 tones were pure tones in octave 5, i.e., in the frequency range from C5 to C6 (523–1046 Hz). These tones were the same as those used by Swanson et al. (2009). As shown in Figures 1, 2, the spectral profiles of the four notes (C5, D5, G5, A5) had peaks on successive electrodes (E20, E19, E18, E17, respectively). Because the ACE filters have broad, bell-shaped frequency responses, each of these pure tones activated multiple electrodes. A large change in fundamental frequency (e.g., C5 to A5) resulted in the activation of a different set of electrodes. A smaller change in fundamental frequency (e.g., C5 to D5) resulted in an overlapping set of electrodes being activated, with changes in the relative amplitude of the pulses on those electrodes, providing an intermediate place-pitch cue (McDermott, 2004).

As explained earlier, because ACE incorporates quadrature envelope detection (Swanson et al., 2007), the filter envelopes (Figure 1) and resulting pulse sequences (Figure 3) had no amplitude modulation, and thus provided no temporal pitch cues (Figure 2).

### Psychophysical Experimental Procedures

Four experimental procedures were used: discrimination, ranking, and two variants of the modified melodies test: backward melodies and warped melodies. These procedures (except for discrimination) were also used by Marimuthu et al. (2016).

#### Discrimination and Ranking

For both the discrimination and ranking procedures, a set of four notes in the same octave were used: {C, D, G, A}. There are six possible pairings of these notes: {CD (2), GA (2), DG (5), CG (7), DA (7), CA (9)}, where the interval in semitones between the notes is given in parentheses. In each trial of the discrimination procedure, the subject was asked to select the note that was different out of four alternatives (e.g., CCAC). In each trial of pitch ranking, the subject was asked whether a sequence of three notes with the first note repeated (e.g., CCA) was either rising or falling in pitch. In both discrimination and ranking, an experimental block comprised 48 trials (six pairings × two orders × four repetitions).

#### Modified Melodies

In each trial of the modified melodies test (Swanson, 2008; Swanson et al., 2009), the name of a familiar melody was displayed to the subject, and its opening phrase was presented twice (in randomized order): once correctly and once with modified pitch. The rhythm was unchanged. The subject was asked to select either the first presentation or the second as the correct version. Trials alternated between two melodies: “Old MacDonald had a farm” and “Twinkle twinkle little star,” which each had a range of nine semitones. The set of interval sizes in the two melodies was {1, 2, 5, 7, 9} semitones; thus, all the intervals larger than one semitone were common to the discrimination and ranking procedures. On each trial, both the correct and modified melodies were transposed by either 0, 1, 2, or 3 semitones, so the total range of notes in a block of trials was an octave.

**FIGURE 4:**
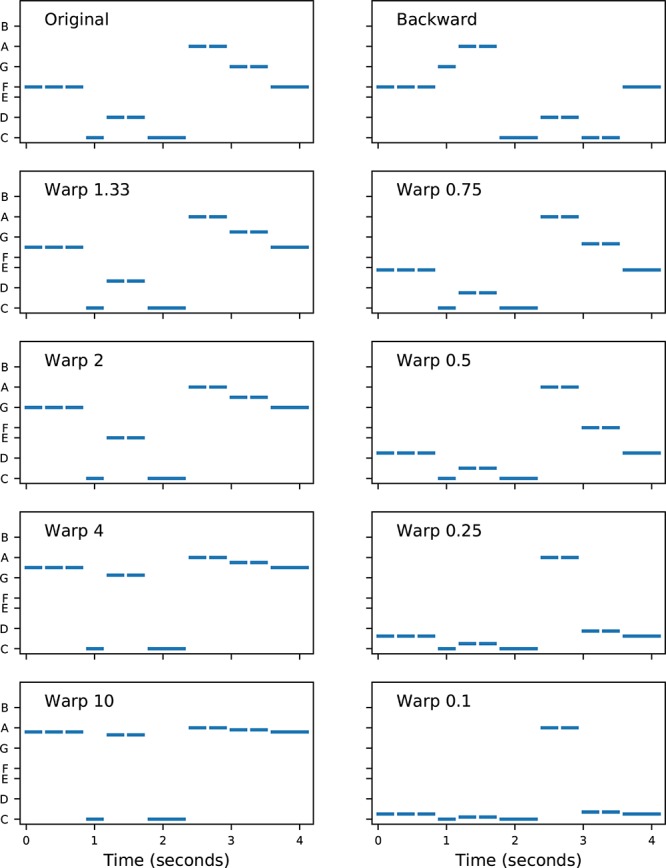
Melodies used in the modified melodies test. Each note is represented by a horizontal line, with length indicating duration and vertical location indicating fundamental frequency. The vertical axis is logarithmic in frequency (i.e., linear in semitones), with note names indicated. Each single note was 300 ms in duration. The **top left panel** shows the original (i.e., correct) melody “Old MacDonald.” The **top right panel** shows the backward melody, which has the same rhythm as the original, but a completely different contour (e.g., the first step in the original is down, from F to C, but the first step in backward is up, from F to G). The remaining panels show the warped melodies. In each warped melody, the highest and lowest notes were unchanged, but the intermediate notes were shifted in pitch according to a “warp factor.” For example, in Warp 2, the intervals in the lower part of the range were doubled in size (e.g., the original two-semitone step up from C to D became a four-semitone step up from C to E), and the intervals in the upper part of the range were halved (e.g., the original two-semitone step down from A to G became a one-semitone step down from A to G#). Conversely, in Warp 0.5, the lower intervals were halved and the upper intervals were doubled. The contour of the warped melodies (i.e., the directions of the steps) was unchanged.

There were two types of pitch modification: backward and warped. Figure 4 displays the original melody of “Old MacDonald” and each of the modified versions used in the study. In backward melodies, the contour of the melody was changed, without changing the set of notes in the melody, by playing the notes in reverse order. Each block comprised 16 trials (two melodies × four transpositions × two repetitions).

In warped melodies, the contour of the melody was maintained, but the sizes of the musical intervals were modified by a “warp factor.” The warp factor determined the amount of expansion or compression of the intervals (refer to Figure 4 for details). A warp factor of 1.0 would leave the intervals unchanged, and hence warp factors further away from 1.0 (either above or below 1.0) had more distorted intervals. A block of warped melodies trials always contained a reciprocal pair of warp factors (in randomized order). All subjects were first tested with the block labeled “W10| 0.1,” in which half the trials were original vs. warp 10, and the other half were original vs. warp 0.1. Subjects were subsequently tested with blocks of trials which progressively increased in difficulty: W4| 0.25, W2| 0.5, and W1.33| 0.75. An informal adaptive rule was applied for each tone type, so that if a subject scored at chance levels, the remaining more difficult blocks were not always tested. Each block comprised 16 trials (two melodies × two warp factors × four transpositions).

### Objectives

The H3 tones were designed to provide temporal cues to pitch, but no place cues. Conversely, the P5 tones were designed to provide place cues to pitch, but no temporal cues. The primary objective of the study was to compare performance between H3 and P5 tones. Many studies have examined CI pitch perception with temporal cues, but few studies have explored musical pitch with place cues. If performance on the discrimination and ranking tasks was comparable between H3 and P5 tones, but performance on the modified melodies test was better with H3 than P5 tones, then it would imply that place cues had more in common with brightness than with true musical pitch.

A secondary objective was to compare performance between H3 and H4 tones. This was of interest because octave 4 (starting at middle C) is very common in music, and normal hearing listeners would be expected to have similar pitch ranking ability for H3 and H4 tones. In contrast, it was hypothesized that CI recipients would have worse performance with H4 than H3 tones, because the upper limit of temporal pitch is typically around 300 Hz, and furthermore the H4 tones exhibited a transition from unresolved to resolved harmonics (Figure 1), yielding a complex mixture of temporal and place cues.

## Results

It was apparent from an initial examination of the results that subject S2 had the lowest scores of any subject for the H3 tones. It was hypothesized that this was because S2 used an ACE map with a channel stimulation rate of 500 Hz in the present study, in contrast to 900 Hz for the other subjects. McKay et al. (1994) recommended that the channel stimulation rate should be at least three to four times the modulation frequency to adequately sample the amplitude modulation waveform. As the primary objective was to compare H3 tones (temporal cues) and P5 tones (place cues), it was decided that the results of S2 should be excluded from any analysis involving temporal cues.

The results were analyzed in several ways. The first analysis applied a Monte Carlo simulation (bootstrap) approach using the binomial distribution (Simon, 1997; Swanson, 2008) to compare scores for H3 and P5 tones (primary objective) and for H3 and H4 tones (secondary objective). As a concrete example, subject S1 had CG discrimination scores of 10 correct out of 16 trials for H3 tones and 15 correct out of 16 trials for P5 tones. The null hypothesis was that the probability of success was the same for H3 and P5 tones. The simulation estimated the probability of observing scores differing by 5 or more if the null hypothesis was true. The best estimate of the null-hypothesis probability (denoted *p*_0_) is the mean score across the two tone types, i.e., *p*_0_ = 25/32 = 0.78. In each simulation run, two random numbers were generated from the binomial distribution, with *n* = 16 (the number of trials), and *p* = *p*_0_ = 0.78. The run was classified as an extreme event if the absolute difference between the two simulated scores was greater than or equal to the difference in the subject’s actual scores (5); this was a two-sided test. The *p*-value for the comparison was estimated as the proportion of extreme events in one million simulation runs, in this case *p* = 0.0526, just missing significance. This approach was extended to examine the scores for a subject across all note pairs. The null hypothesis was that the probability of success varied across the six note pairs, but that at each note pair, it was the same for H3 and P5 tones, yielding a vector of six *p*_0_ values. In each simulation run, six pairs of random numbers were generated using the corresponding *p*_0_ values. The run was classified as an extreme event if the absolute value of the mean difference between the six pairs of simulated scores was greater than or equal to the mean difference in the subject’s actual scores (in this case, 16.7 percentage points). As before, the *p*-value was estimated as the proportion of extreme events in one million simulation runs (in this case, *p* = 0.002). Finally, to examine the group results across the five subjects, the mean scores across tone types yielded a vector of 5 × 6 = 30 *p*_0_ values. Each simulation run generated 30 pairs of simulated scores, and was classified as extreme if the absolute value of the mean difference across the 30 simulated scores was greater than or equal to the mean difference in the 30 actual scores. The per-subject and group results are listed in Table 1.

**TABLE 1:** Results of binomial paired comparisons of scores for H3 vs. P5 tones and H3 vs. H4 tones (excluding subject S2).

**Comparison**	**Procedure**	**Subject**	**Mean difference (%)**	***p*-value**
P5 – H3	Discrimination	S1	16.7	2e-03^∗∗^
		S3	2.1	0.68
		S4	6.2	0.12
		S5	2.1	0.84
		S6	–2.1	0.77
		Group	5.0	0.02^∗^
	Ranking	S1	11.5	1e-02^∗∗^
		S3	5.2	0.13
		S4	5.2	0.20
		S5	10.4	0.11
		S6	–2.1	0.72
		Group	6.0	2e-03^∗∗^
	Modified melodies	S1	–9.4	0.30
		S3	11.9	9e-03^∗∗^
		S4	2.3	0.60
		S5	–0.8	0.91
		S6	–2.1	0.71
		Group	2.0	0.33
H4 – H3	Discrimination	S1	5.2	0.43
		S3	–9.4	0.12
		S4	–15.6	7e-03^∗∗^
		S5	0.0	1.00
		S6	–12.5	0.045^∗^
		Group	–6.5	0.013^∗^
	Ranking	S1	–9.4	0.13
		S3	–25.0	7e-05^∗∗^
		S4	–15.6	6e-03^∗∗^
		S5	4.2	0.61
		S6	–17.7	8e-04^∗∗^
		Group	–12.7	5 e-06^∗∗^
	Modified melodies	S1	–14.1	0.12
		S3	–40.6	1e-05^∗∗^
		S4	–56.2	3e-07^∗∗^
		S5	–10.9	0.015^∗^
		S6	–2.3	0.72
		Group	–19.6	2e-06^∗∗^

*The fourth column shows the mean difference between percent-correct scores, and the fifth column shows whether this difference was significant (under a two-sided test), with an asterisk denoting *p* < 0.05 and two asterisks denoting *p* < 0.01.*

The second analysis was a more traditional ANOVA, but as the results followed a binomial distribution and included many instances of 100% scores, an ANOVA on the percent-correct scores was not considered appropriate. Instead, the scores were first converted into *d*’ sensitivity values (Macmillan and Creelman, 2004). A third analysis applied the non-parametric Friedman test, using the MATLAB Statistics Toolbox (The MathWorks, Inc.). The results for each type of procedure were analyzed separately. The Friedman test is less sensitive than the other tests because it does not consider the size of the differences. For the ANOVA and Friedman analyses, pair-wise differences were subsequently examined with Tukey’s honestly significant difference criterion (using multcompare in MATLAB). The means and comparison intervals were plotted (Figure 8) such that two means differed significantly (*p* < 0.05) if their comparison intervals did not overlap.

### Discrimination and Ranking

**FIGURE 5:**
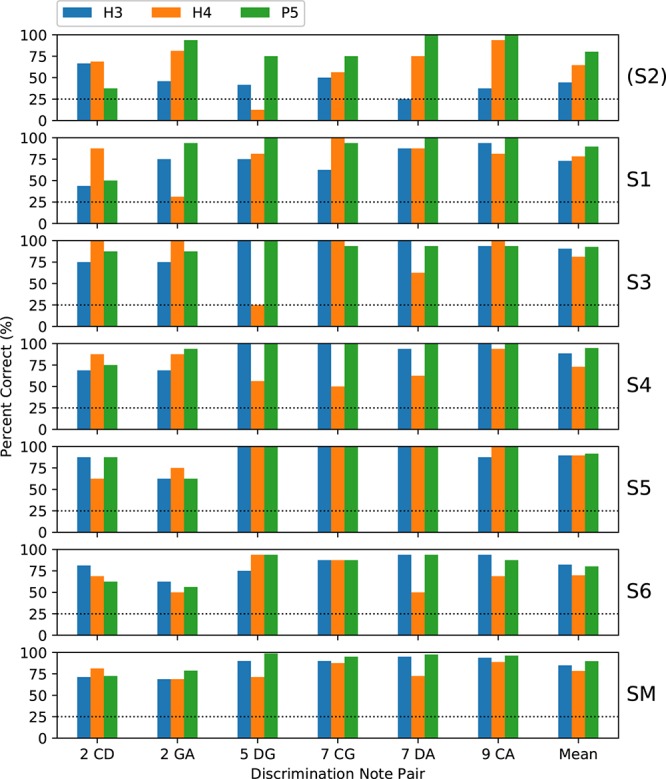
Percent-correct discrimination scores for the three tone types. Each of the **upper panels** shows the scores for a single subject (S1–S6). The **lower panel** (labeled “SM”) shows the group mean scores, excluding subject S2 (who used a 500 pps stimulation rate, unlike the other subjects who used 900 pps). The abscissa labels indicate the note pair, with the interval in semitones preceding the note names, and the right-most set of bars (labeled “Mean”) showing the score averaged across note pairs. The chance score of 25% is indicated by a dotted line.

**FIGURE 6:**
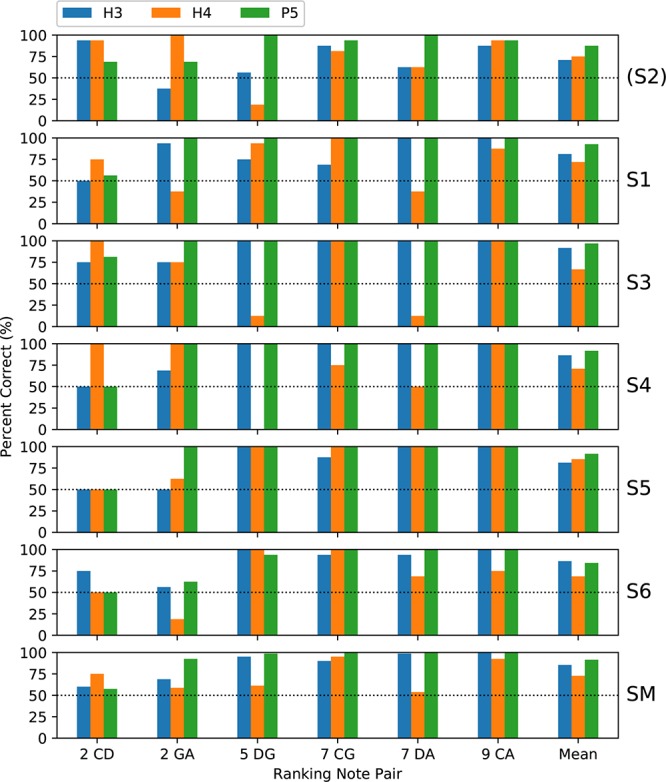
Percent-correct pitch ranking scores, in the same format as Figure 5, except that the chance score is 50%. S2 was again excluded from the group mean.

Subjects completed two blocks of trials for each tone type; except that only one block was performed by S3 for H4 tones and by S5 for all tone types. Figure 5 shows the percent-correct discrimination scores, for each pair of notes, for the three tone types. Similarly, Figure 6 shows the corresponding pitch ranking scores. Scores for subject S2 are shown, but were excluded from the group mean and the statistical analysis. As expected, the overall trend in both procedures was for scores to increase as the interval size increased from two to nine semitones. In our previous study of rate pitch (Marimuthu et al., 2016), the scores were aggregated based on the interval size; however, here the scores for each note pair are reported, because the scores often differed significantly for note pairs that had the same interval (e.g., CG vs. DA 7-semitone ranking scores for H4 tones).

Regarding the primary objective (Table 1), the group mean score was 5 percentage points better with P5 tones than with H3 tones (*p* = 0.02) for discrimination and 6 percentage points better for ranking (*p* = 0.002). For both procedures, the individual subject comparisons only reached significance for one subject (S1). Given that most scores at the larger intervals were near ceiling for both P5 and H3 tones, these mean differences were mainly due to instances such as S3, S4, and S5 scoring 100% for ranking GA for P5 tones, but substantially lower for H3 tones.

Regarding the secondary objective (Table 1), the group mean score was 6.5 percentage points lower with H4 tones than with H3 tones (*p* = 0.01) for discrimination and 12.7 percentage points lower for ranking (*p* < 0.001). Subjects S4 and S6 showed significant differences for discrimination, and subjects S3, S4, and S6 for ranking. The larger difference for ranking (compared to discrimination) was driven by the occurrence of pitch reversals for the H4 tones (Figure 6), i.e., scores significantly worse than chance (50%). Pitch reversals are listed in Table 2 and did not occur for the other tone types.

**TABLE 2:** Pitch ranking reversal scores, and corresponding discrimination scores.

			**Ranking**			**Discrimination**		
**Subject**	**Tone type**	**Note pair**	**Score**	**%**	***p***	**Score**	**%**	***p***
S2	H4	5 DG	3/16	19	0.011^∗^	2/16	12	0.94
S3	H4	5 DG	1/8	12	0.035^∗^	2/8	25	0.63
S3	H4	7 DA	1/8	12	0.035^∗^	5/8	62	0.027^∗^
S4	H4	5 DG	0/16	0	1.5e-05^∗∗^	9/16	56	0.0075^∗∗^
S6	H4	2 GA	3/16	19	0.011^∗^	8/16	50	0.027^∗^

*Scores are given as “number of correct responses/number of trials,” and also as percent correct (%). For ranking, *p* is the probability of obtaining a score as low or lower if the null hypothesis of no perceived pitch difference (*p*_0_ = 0.5) was true. Conversely, *p* for discrimination is the probability of obtaining a score as high or higher if the null hypothesis of no perceived pitch difference (*p*_0_ = 0.25) was true. An asterisk denotes *p* < 0.05 and two asterisks denote *p* < 0.01.*

Because discrimination was a four-alternative forced-choice (4AFC) task, whereas ranking was a 2AFC task, the percent-correct scores for the two procedures should not be directly compared. Instead, the percent-correct scores were converted to *d*’ sensitivity, and a repeated-measures (within subject) ANOVA was performed with factors of procedure (discrimination and ranking), tone type, and note pair (Table 3). There were significant main effects of procedure (*p* = 0.04), tone type (*p* = 0.008), and note pair (*p* = 0.0001). All the interactions of these factors were also significant (*p* < 0.05).

**TABLE 3:** Results of ANOVA analyses of *d*’ sensitivity (excluding subject S2).

**Analysis**	**Factor**	***F*-value**	**Prob > *F***
Repeated measures	Procedure	8.4261	0.0440
ANOVA for discrimination	Tone_type	9.2606	0.0083
and ranking	Note_pair	9.8448	0.0001
	Procedure:tone_type	5.7226	0.0286
	Procedure:note_pair	3.1347	0.0300
	Tone_type:note_pair	2.2808	0.0318
	Procedure:tone_type:note_pair	2.4175	0.0234
Repeated measures	Procedure	6.5584	0.0626
ANOVA for modified	Tone_type	5.9651	0.0260
melodies backward and	Procedure:tone_type	1.6113	0.2582
W10| 0.1			
Repeated measures	Procedure	21.5707	0.0000
ANOVA for H3 tones and	Tone_type	5.0933	0.0870
rate-pitch	Procedure:tone_type	0.8502	0.5564
ANOVA on group mean for	Procedure	9.61	0.0104
all procedures	Tone_type	21.08	0.0019

According to the Friedman test, discrimination scores for the three tone types were not significantly different (*p* = 0.20). The Friedman test showed that ranking scores for the three tone types were significantly different (*p* = 0.017), and pairwise comparisons showed P5 significantly better than H4 (Figure 8).

### Modified Melodies

All of the subjects confirmed that they were familiar with the two melodies from earlier in their life when they had better hearing, but often remarked that neither of the two alternatives in a trial sounded as they remembered the specified melody. Subjects were asked informally to identify the instruments that had played the melodies, and their responses are shown in Table 4. Subjects generally reported that the different tone types sounded like different instruments. Given that the tones were not intended to mimic any specific instrument, there was no “correct” answer, but many responses were common to several subjects, and the responses were generally consistent with the octave range. Interestingly, the most common response for P5 tones was a flute, which has relatively weak higher harmonics compared to other instruments (Schubert and Wolfe, 2006). Almost all responses were wind instruments, most likely due to the temporal envelope of each note, which had a gradual (50 ms) attack and release.

**TABLE 4:** Subjects’ responses when asked to identify the instrument that played the melodies in the modified melodies procedures.

**Subject**	**H3 tones**	**H4 tones**	**P5 tones**
S1	Oboe	Trumpet or oboe	Penny whistle or flute
S2	Saxophone	Clarinet	(Could not say)
S3	Wind instrument: trumpet	Wind instrument	Flute
S4	Oboe	Wind instrument: trumpet	Flute or oboe or clarinet
S5	Oboe	Violin	Flute or clarinet
S6	Bassoon	Clarinet	Woodwind: clarinet

**FIGURE 7:**
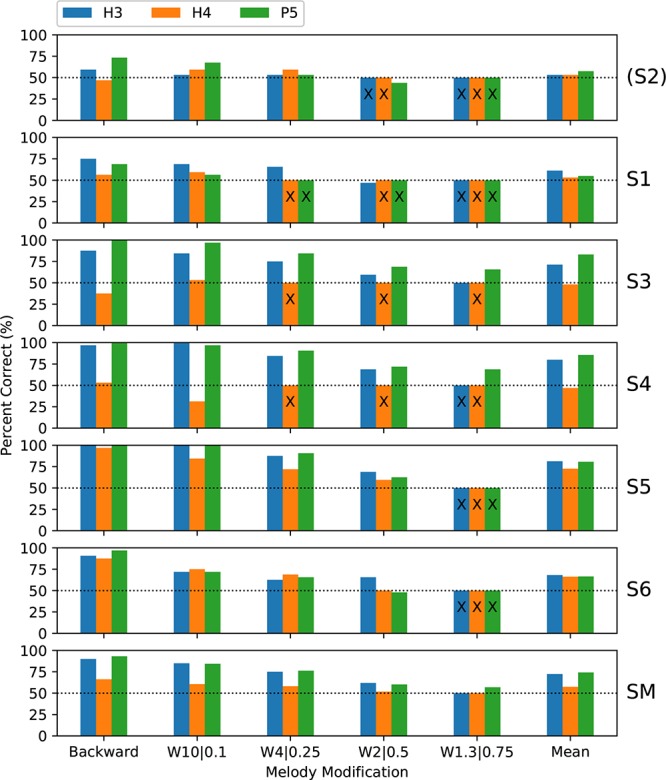
Percent-correct backward melodies scores and warped melodies scores for each subject, and the group mean scores. The format is similar to Figure 6. S2 was again excluded from the group mean. Missing scores for the more difficult warp factors are plotted as chance level (50%) but marked with an “X.”

Subjects completed two blocks of trials for each condition; except that only one block was performed by S3 for H4 backward melodies and by S4 for H3 warped melodies. Figure 7 shows the percent-correct discrimination scores, for backward and warped melodies, for the three tone types. Scores for subject S2 are shown, but were excluded from the group mean and the statistical analysis. Because of the adaptive rule, there were many missing scores for the more difficult warp factors. The missing scores were given an imputed value of 50% (chance level) for visualization purposes (including calculating the mean scores in Figure 7), but are marked with an “X.”

A wider range of performance across subjects was exhibited than with discrimination or ranking, with subjects S1 and S2 rarely scoring above chance, while each remaining subject had some scores at or near ceiling. Scores were similar for backward and W10| 0.1, and the warped melodies scores decreased progressively as the warp factor approached 1.0. Scores for H3 and P5 tones were similar, with lower scores for H4 tones.

The binomial analysis for each subject utilized the backward scores and all the warp scores for that subject that were common to the pair of tone types under comparison. Regarding the primary objective, the group mean scores for P5 tones were 2 percentage points higher than with H3 tones, but the difference was not significant (Table 1). Subject S3 had significantly better scores with P5 tones, by 11.9 percentage points (*p* = 0.009).

Regarding the secondary objective, the group mean score was 19.6 percentage points lower with H4 tones than with H3 tones (*p* < 0.001) (Table 1). All subjects had lower scores with H4 tones, by very large and significant margins for S3, S4, and S5.

**FIGURE 8:**
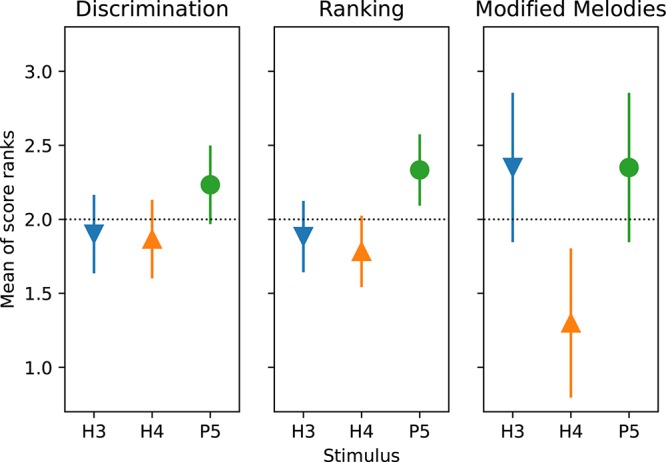
Mean of score ranks (Friedman test) for discrimination, ranking, and modified melodies (backward and W10| 0.1) procedures, for the three tone types. S2 was excluded from the analysis. The dashed horizontal line indicates that if there was no difference between conditions, all would have a mean rank of 2 (mean of {1, 2, 3}). Pair-wise differences were examined with Tukey’s honestly significant difference criterion: two means differ significantly (*p* < 0.05) if their comparison intervals (“error bars”) do not overlap.

The ANOVA statistical analysis only included the scores for the backward and W10| 0.1 modifications, which had no missing data. A repeated-measures (within subject) ANOVA on *d*’ sensitivity was performed with factors of modification type (backward and W10| 0.1) and tone type (Table 3). There was a significant effect of tone type (*p* = 0.026), while the effect of modification type just missed significance (*p* = 0.063). According to the Friedman test, modified melodies scores for the three tone types were significantly different (*p* = 0.019), and pairwise comparisons showed that H4 was significantly worse than both H3 and P5 (Figure 8).

### All Procedures

**FIGURE 9:**
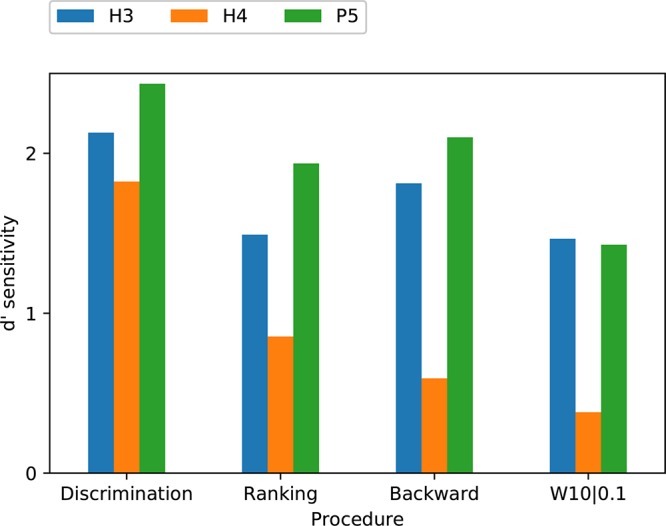
Comparison of group mean *d*’ sensitivity scores for the three tone types across the four procedures. Subject S2 was excluded from the analysis.

To allow an analysis across all procedures, the modified melodies backward and W10| 0.1 percent correct scores were averaged across subjects, and the discrimination and ranking percent correct scores were averaged across both subjects and note pairs. The *d*’ sensitivity scores calculated from these group mean percent-correct scores for each tone type and procedure are shown in Figure 9. The group means were lowest for H4 tones in all four procedures, and highest for P5 tones in three procedures (the exception being W10| 0.1, where P5 and H3 tones had almost equal group means). A two-way ANOVA on *d*’ (Table 3) revealed very significant effects of tone type (*p* = 0.002). Pair-wise comparisons showed that *d*’ was significantly lower for H4 tones than both H3 tones (*p* = 0.008) and P5 tones (*p* = 0.002), with H3 and P5 not differing significantly.

The discrimination task yielded a higher *d*’ sensitivity than the other procedures for all three tone types. The previously mentioned two-way ANOVA on group mean *d*’ (Table 3) also revealed a significant effect of procedure (*p* = 0.01). Pair-wise comparisons showed that *d*’ was significantly greater for discrimination than for ranking (*p* = 0.045) and modified melodies backward (*p* = 0.008), while the comparison between discrimination and W10| 0.1 just missed significance (*p* = 0.07).

## Discussion

### The Centroid Model for Place Cues

Laneau et al. (2004) modeled the place pitch of a CI stimulation pulse sequence by the centroid *c*, calculated as:

(1) $$c=\frac{\underset{k}{\Sigma }\,k\,a\,\left(k\right)}{\underset{k}{\Sigma }\,a\,\left(k\right)}$$

where *k* is the channel number and *a*(*k*) is the amplitude of the corresponding filter envelope. However, this fails to consider the mapping from amplitude to stimulus current. In the ACE strategy, amplitude values that are below a base level are discarded and do not produce a stimulation pulse. These discarded low amplitudes cannot affect the perceived pitch, so they were excluded from the centroid calculation.

**FIGURE 10:**
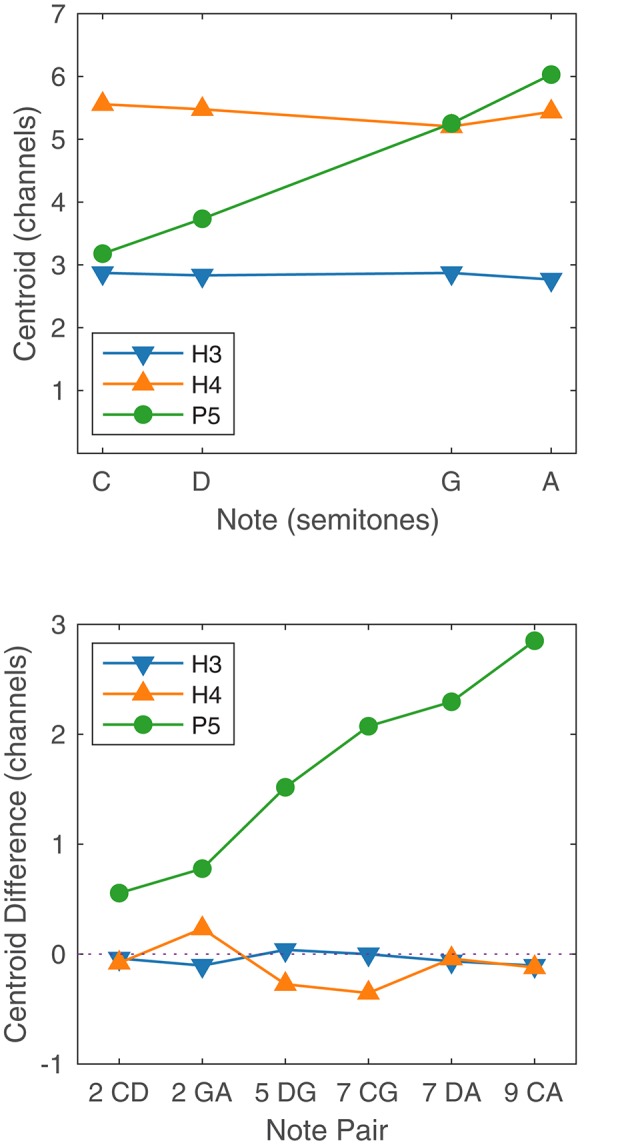
**Upper panel**: Spectral centroid for the four notes C, D, G, and A for each tone type. **Lower panel**: Spectral centroid difference for each note pair, for each tone type. In the abscissa labels, the note names are preceded by the corresponding interval in semitones.

The ability to rank or discriminate two stimuli based on place pitch should depend on the difference between the two centroids. Figure 10 shows the centroids of the four notes (C, D, G, A), for the three tone types, together with the corresponding centroid differences for the six note pairs. Centroids are given in units of channel numbers, e.g., a centroid of 3.5 would mean that the stimulation pattern was centered midway between the third channel (E20) and the fourth channel (E19). The centroids of the four H3 notes were practically identical (just below channel 3, E20), and the centroid differences were negligible, consistent with our earlier claim that there were no place pitch cues for H3 tones.

The horizontal axis of Figure 10 (upper panel) is linear in semitones, with notes C, D, G, and A being located at 0, 2, 7, and 9 semitones, respectively. The centroids of the P5 notes were reasonably close to lying on a straight line, i.e., the centroid was approximately linearly related to the fundamental frequency. Referring to Figure 10 (lower panel), the centroid difference clearly increased with the interval size. If the relationship had been perfectly linear, then the centroid differences for the P5 note pairs CD and GA (both two-semitone intervals) would have been equal, as would those for CG and DA (seven semitones). Instead, the centroid difference for GA was larger than that for CD; and the centroid difference for DA was larger than that for CG. A Monte Carlo binomial analysis (including S2, because only place cues were involved) showed GA group mean scores 14.6 percentage points higher than CD scores for discrimination (*p* = 0.035), and 29.2 percentage points higher for ranking (*p* < 0.001). Thus, the centroid model correctly predicted that the P5 scores for GA would be higher than those for CD, despite both having a two-semitone interval. The centroid model also predicted that the P5 scores for DA would be higher than that for CG, but the differences were not significant because both were near ceiling.

The scores for H4 tones exhibited large variations both between subjects and also between note pairs for the same subject. Scores for the note pairs DG and DA were particularly inconsistent, with subject S5 scoring 100% for both in discrimination and ranking, while the remaining subjects each had instances of low scores. The erratic performance with H4 tones may have been caused by the transition from unresolved to resolved harmonics. Examining the H4 stimuli in Figures 1, 3, it appears that notes C4 and D4 provided primarily temporal cues to pitch, while notes G4 and A4 provided primarily place cues. With the six note pairs delivered in random order in one block of trials, it may have been difficult for subjects to switch attention between place and temporal cues, or confusing to compare a “temporal cue” note with a “place cue” note, such as in the pairs DG and DA.

Furthermore, the place cues for H4 tones were misleading in some cases. The spectral peak of D4 was on E17, while the peak of G4 was more apical, on E18 (Figures 1, 2). Consequently, the centroid of G4 was slightly lower than that of D4 (Figure 10). Thus, although the fundamental frequency increased by five semitones from D4 to G4, the place pitch apparently decreased. In the pair DA, both D4 and A4 had their spectral peak on E17, and had negligible difference in the spectral centroid, and so had much the same place pitch. Referring to Table 2, note pairs DG and DA produced significant pitch reversals for three subjects: S2, S3, and S4. Presumably these subjects were giving more weight to place cues than temporal cues in their ranking judgments. Pitch reversals by CI recipients have also been observed with more natural stimuli such as sung vowels (Sucher and McDermott, 2007; Swanson, 2008; Vandali and van Hoesel, 2012).

### Comparison of Procedures

There are several factors that could contribute to discrimination scores being higher than ranking scores. The ranking task required the subject to order the stimuli along a perceptual scale from low to high, while in the discrimination task, the subject merely had to detect a difference between stimuli, without having to apply any ordering to them. Thus, it is possible that judging the direction of a pitch change was more difficult than detecting a difference in pitch, as has been reported with normal hearing listeners (Moore and Peters, 1992).

Although it was intended that the notes differed only in pitch, it is also possible that there were other unintended differences between the notes. Despite the stimuli having the same acoustic amplitude, it is possible that the loudness of the pulse sequences that were delivered varied between stimuli, as no loudness balancing of the stimuli was conducted. The 4AFC discrimination task has the inherent problem that the researcher cannot be sure that the subject is using pitch to distinguish the stimuli.

A final factor is the presence of pitch reversals. A subject experiencing a pitch reversal is consistently ranking the notes in the wrong order, but can clearly tell the notes apart, and so a high score on the discrimination task for that note pair would be expected. Referring to Table 2, three out of the five pitch reversals had corresponding discrimination scores significantly higher than chance. The decrement in performance of the H4 tones relative to the other tone types was smallest for the discrimination procedure. On balance, it is recommended that the 4AFC discrimination task used here should be avoided in future CI pitch studies, primarily because pitch reversals are not apparent.

The modified melodies test required subjects to decide which of two alternatives best matched their memory of a named familiar melody. Compared to discrimination or ranking, the modified melodies test is more cognitively demanding, so it is not surprising that its scores were lower. Both the contour and the exact interval sizes of familiar melodies are stored in long-term memory (Dowling and Fujitani, 1971). The backward modification required subjects to detect a mismatch in the contour between their memory and each presentation (e.g., a pitch step down instead of up), which in principle only required the ability to rank the pitch of consecutive notes. As might be expected, subjects S2, S3, and S4, who experienced a pitch ranking reversal for the five-semitone DG pair of H4 tones, scored at chance levels for all modified melody conditions with H4 tones.

The warp modification was designed to preserve the melodic contour and change the interval sizes. However, it could be argued that warp factors as extreme as W10| 0.1 did effectively change the contour, as many intervals were compressed into imperceptibly small steps, so that the warped melody was likely perceived to contain long sequences of repeated notes (Figure 4). Therefore, the important question of whether place cues alone can support judgments of musical interval size is best addressed by the scores on the more difficult warp factors. The best performing subjects provided evidence supporting this proposition, with scores on P5 tones significantly above chance by subject S3 for W2| 0.5 (22/32, *p* = 0.025), and by subject S4 for both W2| 0.5 (23/32, *p* = 0.01) and W1.3| 0.75 (22/32, *p* = 0.025).

### Comparison With Previous CI Studies

In our previous study (Marimuthu et al., 2016), the same six subjects performed the procedures of ranking, backward modified melodies, and warped modified melodies as per the present study, but with different stimuli. Each note was a synthetic pulse sequence with a pulse rate equal to the fundamental frequency, so that only rate pitch cues were available. Four spatial stimulation patterns were used, denoted Apex (a single apical electrode), Mid (a single mid electrode), Dual (two electrodes, apical and mid), and Scan (eleven electrodes, from apical to mid). No significant differences were found between scores for the four spatial patterns for pulse rates in the range C3–C4, implying that the strength of the rate pitch percept was independent of electrode place, and of the number of electrodes.

**FIGURE 11:**
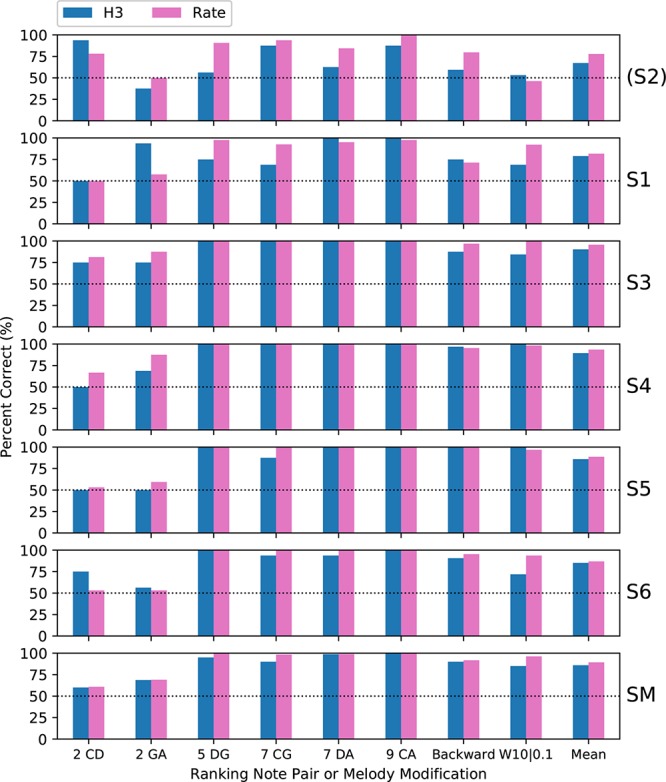
Percent-correct ranking and modified melodies scores for H3 tones, together with corresponding scores for rate-pitch stimuli from a previous study with the same subjects and procedures (Marimuthu et al., 2016). The format is the same as Figure 6. S2 was again excluded from the group mean.

Figure 11 compares the present results for H3 tones with the previous rate pitch results, averaged over the four spatial patterns. The fundamental frequencies of the H3 tones were the same as the pulse rates of the rate pitch stimuli (C3–C4), and the pattern of results was very similar, consistent with other studies that have compared amplitude modulation and pulse rate cues (McKay et al., 1994; Kong et al., 2009).

Subject S2 had the lowest scores of any subject on the H3 tones, and the largest difference (10.5 percentage points) between the H3 and rate pitch mean scores. As mentioned previously, this was most likely because subject S2 used a 500 Hz channel stimulation rate, and so S2 was excluded from the subject mean in Figure 11, and from the following analysis. When converted to *d*’, a repeated-measures (within subject) ANOVA showed no significant effect of stimulus type (*p* = 0.087, Table 1). Thus, the performance with pulse sequences that were amplitude-modulated at F0 was similar to that with pulse sequences with the pulse rate equal to F0. The H3 tones provided temporal cues on nine electrodes, and consistent with Marimuthu et al. (2016), there was no apparent benefit over having temporal cues on a single electrode.

The present results can also be compared to those in another previous study that used the same P5 tones. Results for six CI recipients were reported in Swanson et al. (2009), and one additional subject was reported in Swanson (2008). An earlier version of the modified melodies test was utilized, which included only a single familiar melody (“Old MacDonald”). Four pitch modifications were tested: “Backward” (as in the present study) and “Exchange” altered the melodic contour; while the “Nudge” type preserved the contour, and changed an interval size by either two (“Nudge2”) or five (“Nudge5”) semitones. Scores significantly above chance were obtained by all seven subjects for Backward; by six subjects for Exchange, by four subjects for Nudge5; and by one subject for Nudge2. This is consistent with the results using P5 tones in the present study, where most subjects scored highly for backward, and a small subset of subjects were sensitive to interval size changes in the more difficult warp factors.

### Implications for Cochlear Implant Music Perception

The present study investigated CI pitch perception using the recipients’ own sound processors, with audio signals presented via loudspeaker. This method provides a bridge between real-world listening conditions and psychophysics experiments that deliver synthetic pulse sequences via a research interface.

In Singh et al. (2009), CI recipients used their own sound processors to perform a closed-set melody identification task, with melodies comprised either pure tones or harmonic tones in three F0 ranges: low (104–262 Hz), middle (207–523 Hz), and high (414–1046 Hz). Their upper F0s were identical to that of the three F0 ranges of the present study, although their lower F0s were four semitones lower. In experiment 1, they found better scores with harmonic tones for the middle F0 range than the low F0 range, contrary to the present study, where H4 tones produced worse scores than H3 tones on all procedures. Their harmonic tones had spectral profiles extending up to 4 kHz, so that more higher harmonics would have been resolved, possibly providing more reliable place cues and explaining the difference in results. Alternatively, it may have been due to their recipients using a variety of implant devices and sound processing strategies. Regardless, it appears that tone parameters that would have little impact on pitch scores for normal hearing listeners can have a large impact for CI recipients.

In experiment 2 of Singh et al. (2009), pure tones in the high F0 range produced better scores than harmonic tones in any F0 range, which is consistent with the present study. Singh et al. (2009) labored under the misconception that pure tones would provide good temporal cues, but two of the four recipients in experiment 2 used the Nucleus 24 implant with the ACE strategy, for which the pure tones would have provided no temporal cues. Thus, Singh et al. (2009) inadvertently provided evidence that place cues can support melody identification. One lesson is that knowledge of the algorithms implemented on the sound processor is necessary if it is desired to manipulate or even understand the cues that will be available to a CI recipient.

For some subjects in the present study, performance with pulse rate cues was no worse at octave 4 than at octave 3 (Marimuthu et al., 2016). This suggests that their lower scores with H4 tones, compared to H3 tones, were due to the sound processor failing to provide adequate temporal cues (Figure 2), rather than the temporal cues exceeding the recipient’s upper limit for temporal pitch. This implies that pitch perception for the H4 tones would be improved by a sound coding strategy such as OPAL (Vandali and van Hoesel, 2011, 2012; Vandali et al., 2017, 2018), which enhances temporal cues by providing deeper amplitude modulation over a wider range of F0s.

Finally, it should be remembered that the melodies in the present study consisted of a single note at a time. Even under these ideal conditions, CI recipients’ perception of contour and interval size was worse than that of normal hearing listeners. Real-world music typically comprises multiple instruments playing together, with each instrument often playing chords comprising multiple simultaneous notes. Unsurprisingly, this additional complexity generally results in poor music perception (McDermott, 2004).

### Comparison With Normal Hearing Performance

An informal comparison can be made with normal hearing performance. Frequency discrimination thresholds for pure tones (such as the P5 tones), expressed as a percentage of reference frequency, are less than 1% for listeners with normal hearing (Moore, 1997). F0 discrimination thresholds for harmonic tones with resolved harmonics (such as the H3 and H4 tones) are generally even better (Spiegel and Watson, 1984).

Assuming that an F0 discrimination threshold corresponds to a score of 75% correct on a 2AFC ranking task, then rough estimates of the mean thresholds for the CI recipients in the present study (excluding S2) were 12% (two semitones) for P5 tones, 19% (three semitones) for H3 tones, and 50% (seven semitones) for H4 tones, i.e., more than an order of magnitude worse than normal hearing. It can be inferred that the strength of the pitch perceived by the CI recipients in the present study was relatively weak.

It is informative to compare CI temporal pitch to the pitch of temporal cues alone in normal hearing. Houtsma and Smurzynski (1990) measured normal hearing F0 discrimination thresholds of 2.5–3% for tones containing only unresolved harmonics at F0 = 200 Hz. Kaernbach and Bering (2001) reported that F0 discrimination thresholds for high-pass filtered click trains containing only unresolved harmonics were as low as 1.2% at F0 = 100 Hz, but increased as the filter cutoff frequency increased. This was consistent with earlier results by Cullen and Long (1986), who reported F0 discrimination thresholds for high-pass filtered click trains at F0 = 100 Hz in the range 3–13% across four normal hearing listeners. The best CI recipient in the present study (S3) scored 75% correct for two-semitone intervals (12% F0 change) for H3 tones, which was just in the normal hearing performance range.

### Implications for Models of Normal Hearing Pitch Perception

The relatively good performance on melody tasks using CI place cues is surprising for two reasons. The first reason is the disparities between the place cues in normal hearing and CIs. The frequencies assigned to the CI electrodes do not match the characteristic frequencies of a normal cochlea, and the intermediate place pitch cues depend on the details of sound processing, such as the amplitude roll-off of the filters. Nevertheless, it appears that some recipients can utilize the approximately linear relationship between fundamental frequency and spectral centroid (Figure 10) in the ACE strategy to make judgments of musical interval sizes.

The second reason is that obtaining a musical pitch sensation in the absence of temporal cues has no counterpart in normal hearing. Because neural phase locking is limited to about 5 kHz, pure tones above that frequency provide place cues without temporal cues, but they do not support melody perception (Attneave and Olson, 1971). A sound component that excites a distinct place in the apical to mid region of the cochlea is always accompanied by matching temporal cues: if it has a bandwidth narrow enough to only excite a localized region of the cochlea, then it must resemble a sinusoid, and so the neural firing times will provide a pitch cue.

The goal of a pitch perception model is to predict the pitch that a listener would perceive in response to a given sound. An important aspect to be modeled is the strength of the pitch percepts, which can be quantified by scores on pitch tests. Historically, models of pitch perception have been challenged and refined by applying esoteric sounds that do not commonly occur in the natural environment. Models that use only place cues cannot explain the pitch of a tone containing only unresolved harmonics (Moore and Rosen, 1979), or of amplitude-modulated noise (Burns and Viemeister, 1981). This can only be explained by models that analyze neural firing times (Moore, 1997). Licklider (1951) proposed that an autocorrelation analysis is performed on the neural spike trains at each cochlear place. Meddis and Hewitt (1991) implemented a computer model that summed neural autocorrelation functions across cochlear place, demonstrating accurate predictions of pitch and pitch strength for a wide variety of sounds. Cariani and Delgutte (1996) measured neural firing times in cat auditory nerves, and found that the distributions of inter-spike intervals were consistent with these models.

One issue for autocorrelation models is the absence of physiological evidence for a calibrated neural delay line of up to 30 ms at each cochlear place. Loeb et al. (1983) proposed a spatial cross-correlation model, employing the basilar membrane traveling wave delay instead of a neural delay line. The basilar membrane response to a resolved harmonic shows a large phase shift in the vicinity of the peak. Thus, comparing neural firing times across nearby places in the cochlea yields similar behavior to an autocorrelation model (Shamma, 1985; Carney et al., 2002). Shamma and Klein (2000) showed that such a model can produce a strong response to resolved harmonics, and a weaker response to unresolved harmonics.

To summarize, the most successful pitch models assign a crucial role to neural firing times. Moore and Carlyon (2005) wrote that “a demonstration that pitch can be conveyed purely by place-of-excitation cues would disprove models which propose that timing cues are essential for pitch perception.” It is acknowledged that CI place pitch and CI temporal pitch are extremely impoverished compared to the strong pitch produced by resolved harmonics in normal hearing. However, the pitch of CI temporal cues resembles the pitch of unresolved harmonics in normal hearing, and although weak, both support judgments of the size of musical intervals, and the recognition of melodies, and both are widely accepted to be true musical pitch (Moore and Carlyon, 2005). The present study suggests that CI place cues provide pitch of similar strength to CI temporal cues, and support similar levels of melody recognition. Hence, the present results imply that a truly comprehensive pitch model should not only produce a weak pitch percept for temporal cues in the absence of place cues, it should also produce a weak pitch percept for place cues in the absence of temporal cues.

An alternative explanation is that the recipients in this study perceived the CI place cues as brightness, consistent with the spectral centroid model of brightness in normal hearing, but that they could utilize brightness to score well on the modified melodies test. McDermott et al. (2008) reported a set of experiments showing that normal hearing listeners were able to recognize patterns in brightness (and loudness). In each trial, subjects heard a randomly-generated five-note sequence, followed by a transposed sequence, and were asked whether the contours of the two sequences were the same or different. The notes in a sequence varied in either pitch, brightness, or loudness. Subjects performed best when both sequences were pitch sequences, but scores were still well above chance when both were brightness sequences, or when the two sequences were of different types (e.g., a pitch sequence compared to a brightness sequence). In the final experiment, subjects were presented with familiar “melodies” played as either pitch, brightness, or loudness sequences, and asked to name them. Performance with brightness and loudness sequences was well above chance, and was similar to performance with pitch sequences where all the intervals had been stretched by a factor of two. The backward melodies in the present study had an incorrect contour, so it is possible that subjects could utilize brightness cues to identify them.

In a later study (McDermott et al., 2010), subjects were presented with two pairs of notes differing in either pitch, brightness, or loudness, and asked which of the two intervals was wider. Subjects were able to make judgments of interval sizes for brightness (and loudness), although the thresholds for brightness interval size, measured in semitones, were twice as large as thresholds for pitch interval size (i.e., subjects were less accurate for brightness than pitch). However, the experiments in McDermott et al. (2010) did not provide a musical context for the intervals. The warped melodies in the present study had incorrect interval sizes, but merely being able to judge which of two isolated intervals was larger would not be sufficient to score well; subjects had to decide which melody had the musically-correct intervals. In summary, it is not clear whether brightness cues could explain the scores of the CI recipients in the present study; evaluating normal hearing listeners on the modified melodies test with brightness sequences may help to resolve the issue.

McDermott et al. (2008) did not provide much in the way of subjective reports. Although subjects could recognize that a particular loudness sequence had the same up-and-down pattern as the pitch changes in a familiar melody, this could simply reflect general pattern-matching cognitive abilities, and it seems unlikely that anyone would claim that they could “hear a melody” in a pattern of loudness changes (Moore and Rosen, 1979). Because brightness scores were similar to loudness scores in McDermott et al. (2008), the same may be true of brightness contours. Ultimately, we must rely on the CI recipients to tell us whether they could hear a melody in the P5 tone sequences. Based on their subjective reports (Table 4), it seems that they did. Subjects readily described the P5 melodies as being played by wind instruments, just as they did with the H3 and H4 melodies; indeed, the labels of oboe and clarinet were applied to all three tone types. There was no indication that the P5 tones provided a different type of perceptual experience to the H3 tones.

## Conclusion

Cochlear implant pitch perception was measured using discrimination, ranking, and the modified melodies test. Group mean scores for H4 tones were significantly poorer than H3 tones, most likely because of inadequate temporal cues and misleading place cues. Some subjects experienced pitch reversals with H4 tones, eliminating any ability to perceive melodies. Group mean scores for P5 tones (place cues alone) were at least as high as those for H3 tones (temporal cues alone). The scores with P5 tones were qualitatively consistent with a centroid model of place pitch perception. Despite the similarity to the centroid model for brightness in normal hearing, the results suggest that CI place cues can provide a sense of musical pitch, albeit much weaker than that provided by pure tones in normal hearing.

## Data Availability Statement

The datasets generated for this study are available on request to the corresponding author.

## Ethics Statement

The studies involving human participants were reviewed and approved by the Macquarie University and Sydney South West Area Health Service Human Research Ethics Committees. The patients/participants provided their written informed consent to participate in this study.

## Author Contributions

All authors contributed to the conception and design of the study. BS wrote the experimental software and the first draft of the manuscript. VM tested the subjects. BS and VM performed the statistical analysis, contributed to manuscript revision, and read and approved the submitted version.

## Conflict of Interest

BS is an employee and shareholder of Cochlear Ltd., manufacturer of the Nucleus^®^ cochlear implant system. VM was previously a part-time employee of Cochlear Ltd. The remaining author declares that the research was conducted in the absence of any commercial or financial relationships that could be construed as a potential conflict of interest.
